# Special Issue: “Genetic Counseling and Genetic Testing in Precision Medicine”

**DOI:** 10.3390/jpm13081192

**Published:** 2023-07-27

**Authors:** Erin Turbitt, Chris Jacobs, Alison McEwen

**Affiliations:** 1 Discipline of Genetic Counselling, University of Technology Sydney, Ultimo, NSW 2007, Australia; chris.jacobs@surrey.ac.uk (C.J.); alison.mcewen@uts.edu.au (A.M.); 2 School of Health Sciences, University of Surrey, Guildford, Surrey GU2 7XH, UK

## 1. Special Issue Overview

Progress in genomic technologies has spurred innovation in healthcare and medicine, contributing to improved health and well-being. These improvements have been made possible by way of precision medicine, which is defined as an approach that incorporates an individual’s genomic, environmental, and lifestyle information to guide decisions related to their medical management [1]. Delivering on the promises of precision medicine comprehensively, equitably, and promptly depends on optimizing health service delivery to facilitate access to timely, evidence-based genetic and genomic testing. Improvements in access to genetic counseling and testing enable the delivery of the ‘right care to the right patient at the right time’ [2]. This Special Issue of the *Journal of Personalized Medicine* showcases the latest research on genetic counseling and genetic testing in precision medicine.

The Special Issue includes 14 articles from four countries including Australia, Switzerland, Spain, and the United States. The articles demonstrate the breadth of genetic counselors’ practice, the variety of specialty areas and testing types, and a range of research participants’ perspectives. Pleasingly, to tackle this topic, researchers are using a mix of methods, with articles in this Special Issue including qualitative, quantitative, systematic review, and mixed-methods studies. Co-design methods are also represented in this special issue [3,4]. Co-design research methods play an important role in research into genetic service delivery by promoting collaboration with patients or clients with lived experience, as well as other stakeholders including clinicians. By involving such stakeholders, co-design helps ensure that research is relevant, patient-centered, and impactful.

## 2. Expanding Access to Genetic Counseling and Genetic Testing

Articles in the Special Issue highlight the ever-evolving and expanding ways that people are obtaining genetic counseling and genetic testing. Traditionally, these genetic services were only available in specialized tertiary healthcare settings for those with suspected genetic conditions or a family history of such conditions. However, new approaches to accessing genetic counseling and genetic testing are being investigated. One such approach involves offering research participants actionable secondary findings through their participation in a genomic research study [5]. This means that individuals taking part in the study can receive information about additional genetic risks beyond the specific focus of the research, broadening the scope of genetic information that can be provided to participants. Another avenue being explored is the screening of healthy individuals in the community for genetic risk factors associated with melanoma [6]. The third approach represented in this Special Issue is providing genetic counseling and testing through oncology centers, facilitated by oncology nurses [7]. This integrated approach allows individuals at risk of cancer to access genetic counseling and testing within the context of their cancer care. Integrating genetic services into oncology settings helps address the increasing demand for genetic healthcare among individuals concerned about cancer risks.

While the majority of articles focus on genetic healthcare for individuals at risk of cancer, it is worth noting that another area experiencing significant growth in genetics is reproductive genetic carrier screening (RGCS) [4]. RGCS involves using genetic testing to assess the risk of genetic conditions in individuals planning to have children. By identifying potential genetic risks prior to conception, individuals and couples can make informed decisions about their reproductive options. A scoping review published in the Special Issue identified the genetic counseling needs of individuals undergoing RGCS [8]. The researchers found a need for further research on the genetic counseling requirements in large-scale RGCS programs, as existing knowledge mostly focuses on smaller programs. A difficulty lies in addressing potential conflicts between commercial and patient interests. One article in the Special Issue contributes towards filling this gap identified by Edwards et al. [9]. This paper details the protocol of the Australian reproductive genetic carrier screening project Mackenzie’s Mission to generate implementation evidence to inform a national program [9].

## 3. Assessing Patient-Reported Outcomes in Genetics

The importance of assessing patient-reported outcomes of genetics and genomics in a range of contexts is increasingly being recognized. Three articles in this Special Issue contribute to these efforts [4,5,10]. The contexts in the Special Issue that include patient-reported outcomes are (1) when participants are receiving unexpected (secondary) findings from genomic research, (2) when individuals are undergoing reproductive genetic carrier screening, and (3) for parents who receive their children’s results in an undiagnosed disease program. Across these three contexts, many of the outcomes reported can be categorized as personal utility (non-medical outcomes), including increased understanding of their (or their child’s) condition, improved knowledge, and reproductive empowerment. Clinical outcomes were noted for patients receiving unexpected findings from their participation in a research study: participants underwent confirmatory genetic testing, engaged in breast cancer risk management strategies, and informed their family members about the genetic information. The link between informing patients of their genetic risk, and the subsequent actions (behaviors) is another key area explored in this Special Issue.

## 4. The Importance of Behavior Change Techniques

Simply providing individuals with their genetic test results often does not lead to behavior change. Behavior change techniques and theory provide evidence-based strategies to effectively engage and motivate people. By integrating behavior change techniques and theory into the process of delivering genetic test results, health professionals can enhance individuals’ ability to implement positive behavior changes based on their genetic information. The role of both genetic counselors and oncology nurses in facilitating behavior change was highlighted in this Special Issue [7,11]. Jacobs et al. found three behaviors that genetic counselors aimed to facilitate in their clients: increasing appointment attendance, promoting access to information, and encouraging the sharing of genetic information with family members [11].

## 5. Communicating Complex Information to Clients and Their Family Members

Three papers in the Special Issue highlighted the importance of family communication [12,13,14]. One of these articles introduces the concept of the communication chain in the context of disclosing genetic test results to clients [12]. Pedrazzani et al. described the first link in this chain, which takes place during the post-test session where clients learn their genetic information. It is crucial to convey this information in a manner that clients can comprehend easily, take appropriate action upon, and uses their preferred language. The articles in this Special Issue underscore the importance of achieving these objectives to ensure the adoption of desired behaviors [15,16]. The second link in the communication chain that Pedrazzani et al. reported involves clients sharing their genetic information with family members. Both Pedrazzani et al. and Poulton et al. discussed the challenges associated with this second link of the communication chain as well as the potential role of genetic counselors facilitating clinician-mediated family communication. Similarly, Sarki et al. found that family members are more likely to undergo genetic testing if recommended by their clinician.

## 6. Summary

In establishing the direction of the Special Issue of the *Journal of Personalized Medicine,* we chose to focus on rigorous research by and for genetic counselors with the support of other relevant health professionals and academic fields. The majority of the articles include genetic counselor researchers leading or co-authoring the research, demonstrating clear evidence of continued growth of researcher capability. As a multi-disciplinary editorial team, we celebrate the diverse research showcased in the Special Issue and call for further development of a rigorous evidence base for genetic counseling and testing. This is particularly important as we enter a time where genomic healthcare and precision medicine expand into specialty areas where genetic and genomic testing is likely to be facilitated by healthcare professionals with limited access to genetic counselor support.
